# Enhancing the Yield of Bioactive Compounds from *Sclerocarya birrea* Bark by Green Extraction Approaches

**DOI:** 10.3390/molecules24050966

**Published:** 2019-03-09

**Authors:** María de la Luz Cádiz-Gurrea, Jesús Lozano-Sánchez, Álvaro Fernández-Ochoa, Antonio Segura-Carretero

**Affiliations:** 1 Department of Analytical Chemistry, University of Granada, C/Fuentenueva s/n, 18071 Granada, Spain; jesusls@ugr.es (J.L.-S.); alvaroferochoa@ugr.es (Á.F.-O.); 2 Research and Development of Functional Food Centre (CIDAF), PTS Granada, Avda. Del Conocimiento s/n., Edificio BioRegion, 18016 Granada, Spain

**Keywords:** *Sclerocarya birrea*, bark, green extraction, proanthocyanidins, PLE, SFE

## Abstract

*Sclerocarya birrea* is a tree indigenous to Southern Africa with significant importance in rural livelihoods for food, medicine, and carving. The bark, which contains 10–20% tannin, provides several pharmacological benefits as an antidiabetic, anti-inflammatory, antimicrobial, anti-atherogenic, and antioxidant medication, among others. This study compared different extraction techniques used to recover bioactive compounds from marula bark. For this purpose, solid–liquid extraction, supercritical fluid extraction (SFE), and pressurized liquid extraction (PLE) were performed under selected conditions, using only “food-grade” solvents. The potential use of the proposed extraction methodologies was evaluated in term of yield, and the individual phenolic composition determined by HPLC–ESI–TOF–MS. PLE provided a high extraction yield in all experimental conditions. With regard to bioactive compounds composition, a total of 71 compounds, a significant percentage of which in a galloyl form, were distributed in five major categories. The largest number of compounds, mostly flavonoid aglycones, were extracted by PLE, generally when the extraction was developed at low temperatures. SFE did prove effective as a way of extracting antidiabetic proanthocyanidins. Advanced extraction techniques represent a powerful tool to obtain bioactive compounds from *S. birrea* bark, which can be used as supplements or food ingredients, promoting the valorization of this crop.

## 1. Introduction

*Sclerocarya birrea*, also known as marula, is a medium-sized to large deciduous Savannah tree indigenous to Southern Africa belonging to the family Anacardiaceae. It is favored by wildlife in conservation areas and is of significant importance in rural livelihoods [1]. In addition, it is one of the plants that played a role in feeding people in ancient times and was dubbed “food of kings”. The amounts produced in recent years are surprising (at least considering that this is a “lost” crop and as yet, is moderately grown in organized production). For instance, about 500 tons of marula were commercially processed for juice, and 2000 tons for liqueur just in South Africa [2]. The bark, which contains 10–20% tannin as well as traces of alkaloids, provides fiber and gum to produce ink or red dye [3]. A decoction of the bark has been used to treat dysentery, diarrhea, rheumatism, and as a prophylactic remedy against malaria [4]. Moreover, several studies about *S. birrea* steam bark extracts have reported their pharmacological properties such as antidiabetic [5,6], anti-inflammatory [7,8], antimicrobial [9,10], anti-atherogenic [11,12], and antioxidant [13,14], among others.

A comprehensive phytochemical analysis of non-selective *S. birrea* bark extractions using reversed-phase high-performance liquid chromatography (RP-HPLC) coupled to mass spectrometry (MS) and tandem MS through a hybrid mass analyzer quadrupole-time-of-flight (QTOF) and with atmospheric-pressure ionization techniques as electrospray ionization (ESI) has been previously reported by Jiménez-Sánchez et al. This methodology showed a very high degree of galloylation, which may have a role in the bioactivity attributed to these extracts [15]. Recently, proanthocyanidins, a group of naturally occurring polyphenolic bioflavonoids, have attracted great interest for their potentially beneficial vasodilator, anti-carcinogenic, anti-allergic, anti-inflammatory, anti-bacterial, cardioprotective, immune-stimulating, anti-viral, and estrogenic activities [16].

Since matrixes of plant origin contain thousands of diverse metabolites of varying polarities and concentrations, it is difficult to develop a single method for the optimum extraction of all metabolites. Within this context, different extraction techniques have been used to obtain phenolic compounds contained in barks, being conventional extraction methods such as solid–liquid extraction (SLE) [13,17,18] the most employed. Nowadays, the search for an extraction methodology to obtain phenolic compounds is the focus of numerous studies, which have the objective to find more cost-effective and greener techniques to obtain extracts with large amounts of bioactive compounds. Among such technologies, supercritical fluid extraction (SFE) using CO_2_ and pressurized liquid extraction (PLE) are the most widely employed to obtain bioactive components from natural sources, being effective not only as laboratory tools but also for agri-food industries [19,20,21,22]. 

Therefore, the main objective of this study was to compare different extraction methodologies such as conventional solid–liquid extraction, SFE, and PLE, performing under selected conditions and using only “food-grade” solvents like water and ethanol, for the extraction of phenolic compounds from *S. birrea* bark. In order to do a comprehensive characterization of the phenolic profile of the obtained extracts, a method based on HPLC–ESI–TOF–MS was used.

## 2. Results and Discussion

### 2.1. Characterization of Bioactive Compounds from S. birrea by HPLC–ESI–TOF–MS 

**Figure 1:**
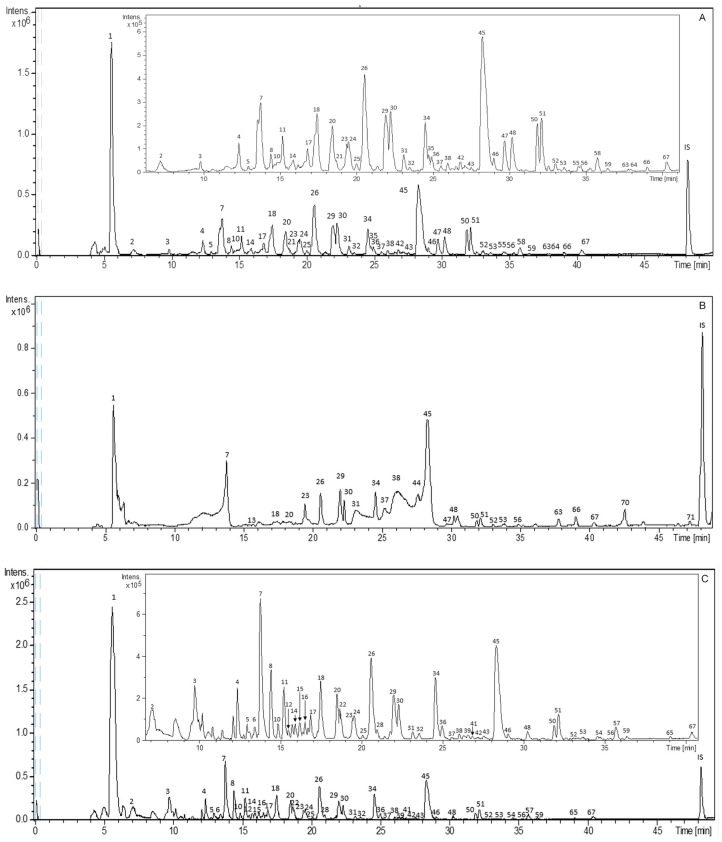
Base Peak Chromatograms (BPCs) of representative *Sclerocarya birrea* extracts obtained with different extraction techniques: (**A**) solid–liquid extraction (SLE); (**B**) supercritical fluid extraction (SFE), and (**C**) pressurized liquid extraction (PLE). Peak numbers correspond to those of Table 1.

Figure 1 shows the base peak chromatograms (BPCs) obtained in negative polarity for representative SLE, SFE, and PLE extracts. In addition, all the identified compounds are presented in Table 1, numbered according to their elution order. This table includes the complete information obtained by HPLC–ESI–TOF–MS analysis and a list of extracts in which the proposed compounds have been detected. All these compounds were characterized by the interpretation of their mass spectra provided by the TOF mass analyzer and the information previously reported. 

In the present study, 71 compounds distributed in five major categories (gallic acid and derivatives, monomers from (epi)catechin and derivatives, dimers from (epi)catechin and derivatives, flavonoids, and other compounds) were tentatively identified. The reported results pointed out that a significant large percentage of them appeared in a galloyl form. Four compounds remained unknown, as indicated in Table 1.

#### 2.1.1. Gallic Acid and Derivatives

Concerning the gallic acid and derivatives group, seven compounds were found in the extracts. These compounds corresponded to galloyl glucose isomers (peaks 4 and 5) at *m*/*z* 331, which eluted earlier than gallic acid (peak 7) at *m*/*z* 169 because of the presence of a sugar molecule in their structures. In addition, four galloyl derivatives were also detected at different retention times depending on their apolar substituents as methoxy groups: dimethoxy-hydroxyphenyl-*O*-galloyl glucopyranoside, hydroxyl-methoxyphenyl-*O*-galloyl glucopyranoside, galloyl-glucosyl dihydroxymethoxyacetophenone, and trihydroxystilbene glucosyl-*O*-gallate at *m*/*z* 483, 453, 495, and 541, respectively (peaks 28, 32, 39, and 56). Among them, peaks 28 and 32 have previously been mentioned in the literature for other barks [23,24,25], but this is the first time that they were detected in *S. birrea*. 

#### 2.1.2. Monomers and Dimers from (Epi)Catechin and Derivatives

Twelve compounds were detected as monomers from (epi)catechin and derivatives. Among them, peaks 23 and 34 yielded deprotonated molecules at *m*/*z* 289, being identified as catechin and epicatechin, respectively. Moreover, different gallate derivatives were identified as (epi)gallocatechin isomers at *m*/*z* 305 (peaks 10 and 22), (epi)gallocatechin gallate isomers at *m*/*z* 457 (peaks 19, 29, and 35), (epi)catechin gallate isomers at *m*/*z* 441 (peaks 44, 45, and 48), (epi)catechin glucoside gallate at *m*/*z* 603 (peak 46), and (epi)afzelechin gallate at *m*/*z* 425 (peak 52). 

The largest class of compounds in all the extracts from *S. birrea* comprised compounds that were mainly esterified with gallic acid and composed of dimers from (epi)catechin. When procyanidins incorporate gallate in their structure, they show an increase of their antiradical power until polymerization degree equal to 3 [26]. In *S. birrea* extracts, these compounds were identified as procyanidin B-type isomers at *m*/*z* 577 (peaks 14 and 47) and other gallate derivatives such as procyanidin dimer gallate isomers at *m*/*z* 729 (peaks 25, 26, and 51). Among others gallate derivatives, we could also detect a gallo(epi)catechin dimer at *m*/*z* 609 (peak 6) and two isomers from bis(epi)gallocatechin monogallate (peaks 9 and 11) at *m*/*z* 761. A dimeric proanthocyanidin with two (epi)gallocatechin units and two galloyl residues (peak 17) was found at *m*/*z* 913. Moreover, two isomers at *m*/*z* 897 from (epi)gallocatechin gallate (epi)catechin gallate (peaks 24 and 42), six isomers with different elution behavior at *m*/*z* 745 from (epi)gallocatechin gallate (epi)catechin (peaks 12, 16, 18, 20, 21, and 38) and (epi)gallocatechin (epi)catechin (peak 15) at *m*/*z* 593 were identified in the extracts. These two last isomers (*m*/*z* 745 and 593) correspond to a loss of a gallic acid residue (−152 Da) from the earlier one, respectively (*m*/*z* 745 from 897 and *m*/*z* 593 from 745). An (epi)gallocatechin (epi)catechin gallate (peak 41) was also detected at *m*/*z* 743. In the end, the HPLC–ESI–TOF–MS method allowed to identify four isomers of the (epi)catechin gallate dimer at *m*/*z* 881 (peaks 30, 31, 37, and 50).

#### 2.1.3. Flavonoids

Regarding flavonoids (not derived from (epi)catechin), 13 compounds were detected in *S. birrea* extracts and identified as flavanols, flavanones, flavanonols, and chalcones. Among these, the flavanol subclass was the most abundant one. The phenolic compounds belonging to this class were dihydromyricetin isomers at *m*/*z* 319 (peaks 49 and 57), myricetin glucoside at *m*/*z* 479 (peak 53), jaceidin triacetate at *m*/*z* 485 (peak 54), rhamnetin at *m*/*z* 315 (peak 61), and dihydroquercetin and quercetin glucoside at *m*/*z* 303 and 463 (peaks 62 and 64, respectively). Concerning flavanones, three compounds were also detected, two of them at *m*/*z* 271 (peaks 68 and 71), and the third one identified as eriodictyol glucoside at *m*/*z* 449 (peak 36). With regard to flavanonol, compound 69 had a deprotonated molecule at *m*/*z* 303 and was tentatively characterized as taxifolin. Within the chalcone sub-class, two chalcones, which were identified as phloretin-C-glucoside and di-C-glucoside, were found at *m*/*z* 435 and 597 (peaks 60 and 55). In contrast to the ubiquitously present flavonoids, dihydrochalcones seem to be restricted to about 30 plant families [27].

#### 2.1.4. Other Compounds

Other phenolic compounds not belonging to the previous groups were tentatively identified as hydroxylbenzoic acids derivatives (peaks 13, 27, and 65), homaloside D (peak 59), and ellagic acid (peak 67).

Finally, other identified compounds were a cyclic polyol identified as quinic acid (peak 1) at *m*/*z* 191 and sugars (peaks 2 and 3). In addition, a lignan glycoside identified as lyoniside was also detected at *m*/*z* 551 (peak 43). Concerning stilbenes, two isomers at *m*/*z* 329 were tentatively identified as pentamethoxystilbene (peaks 63 and 66). These compounds were reported to show a high anti-proliferative effect on different human cancer cell lines [28,29,30]. Moreover, the dicarboxylic fatty acid corresponding to deprotonated molecule at *m*/*z* 187 was identified as nonanedioic acid (peak 70). There were some compounds whose structure could not be identified when the MS experiment was performed (peaks 8, 33, 40, and 58).

### 2.2. Effect of Conventional and New Extraction Techniques on Bioactive Compounds Recovery

#### Extraction Yield

The extraction yields by weight (extract weight/initial weight, %) for all experiments are reported in Table 2. It appears clear that PLE represents the best option for the extraction, since this technique provides a high extraction yield in all experimental conditions. In this sense, the highest yield values were obtained from PLE-I and -G, when the temperature was set to 200 and 176 °C, and the solvent was ethanol/water 50:50 and 85:15 (*v*/*v*), respectively (Table 3). These results pointed out that an increase in both the temperature and the percentage of ethanol in the solvent may favor a faster migration of the components from the sample to the solvent, reaching higher yield values. 

Concerning the SLE methodology, it gave lower yields than PLE, providing the highest recovery when the proportion of ethanol/water was set to 50% (SLE-C). These results are in agreement with previous reports which have established that the application of solvent mixtures enhance the extraction yields by improving the solubility and increasing the interaction of the targeted analyte with the extraction solvent [31]. As regards SFE, this technique showed the worst efficient extraction yield.

### 2.3. Comparative Study of the Phytochemical Composition of S. Birrea Extracts Obtained with different Extraction Methodologies

**Figure 2:**
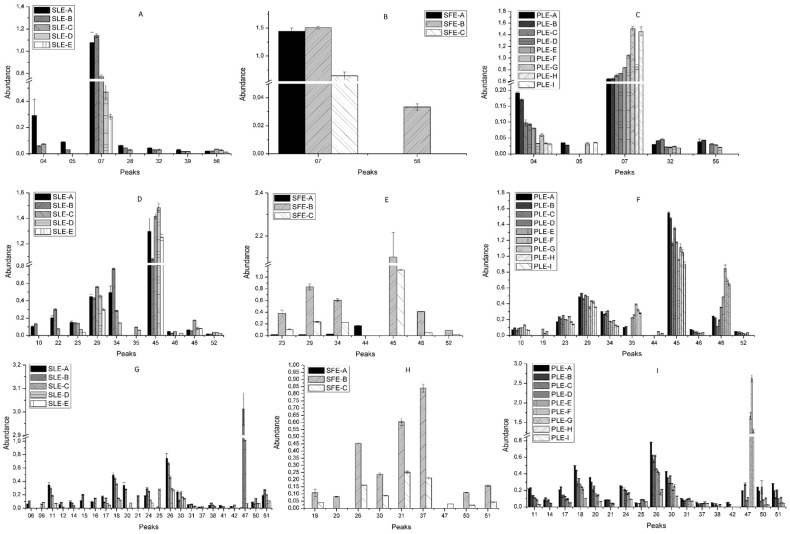
Abundance * of the different groups of compounds: gallic acid and derivatives (**A**–**C**), monomers of flavan-3-ols and derivatives (**D**–**F**), dimers of flavan-3-ols and derivatives (**G**–**I**) extracted from *S. birrea* by SLE (**A**,**D**,**G**), SFE (**B**,**E**,**H**), and PLE (**C**,**F**,**I**). * means of the comparison of the ratio of the analyte peak area to the internal standard peak area.

**Figure 3:**
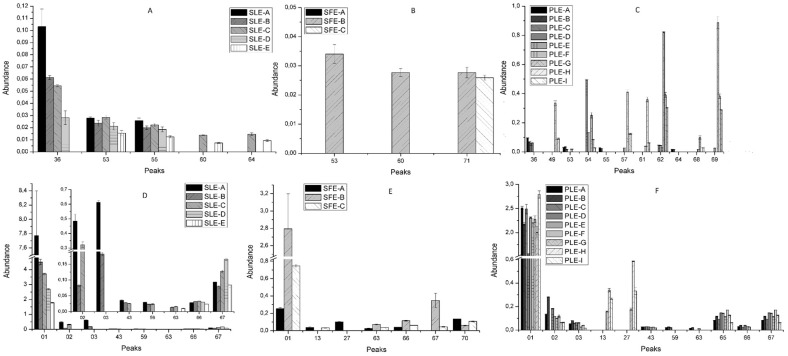
Abundance * of the different groups of compounds: non-derivatives flavan-3-ols flavonoids (**A**–**C**) and other compounds (**D**–**F**) extracted from *S. birrea* by SLE (**A**,**D**), SFE (**B**,**E**), and PLE (**C**,**F**). * means of comparison of the ratio of the analyte peak area to the internal standard peak area.

The differences in the chemical structure of the phenolic compounds confer differences to their physicochemical properties such as solubility, thermal and chemical stability, and ability to link to other compounds. Moreover, the molecular weight and conformational flexibility of phenols have been reported to influence their tendency to be retained in the plant cell wall matrix when an extraction approach is applied [32]. In this scenario, the technological processes used to extract *S. birrea* bark were evaluated to determine compositional variations. The base peak area of each signal in HPLC–MS chromatograms was used to provide semi-quantitative information for comparison purposes. These differences are shown in Figure 2 and Figure 3 by abundance as means (Tables S2–S4) of comparison of the corrected area of each individual compound (analyte peak area/internal standard area). The phenolic contents showed a large variation among the different extracts according to the extraction technique employed and the different conditions of each extraction system. 

Figure 2A–C show the recovery of the gallic acid and derivatives group with different extraction techniques and experimental conditions. In this group, the most abundant extracted compound with the three applied methodologies was gallic acid (peak 7), being the yields of green technologies (SFE and PLE) significantly higher than those obtained with SLE. On the contrary, this conventional technique showed the greatest number of different compounds belonging to this sub-class. Almost all SLE-extracted compounds showed a positive recovery trend with the increase of the water percentage, that is, the higher the water percentage, the more abundant the compound recovery, except for trihydroxystilbene glucosyl-O-gallate (peak 56). For the latter compound, the most efficient SLE condition was set to 50:50 EtOH/H_2_O (*v*/*v*). 

Regarding the SFE extracts, gallic acid (peak 7) was obtained in the three experimental conditions, although there were significant differences between SFE-C (lower values) and the other experiments developed using this technique (Table S5). Furthermore, trihydroxystilbene glucosyl-*O*-gallate (peak 56) was only extracted under the SFE-B condition. This condition differs from SFE-A in the use of ethanol as supercritical fluid co-solvent and from SFE-C in the use of ethanol in the sample preparation. The combination of both factors could be responsible for the presence of this compound in SFE-B extracts. 

In the case of PLE, only galloyl glucose isomer and gallic acid (peaks 4 and 7, respectively) were detected in all conditions but with a different behavior. The galloyl glucose isomer showed high abundance values at low temperature. With regard to gallic acid, this compound showed the opposite performance, being the glucose unit the difference between these compounds. Moreover, the other glycosylated compounds (peaks 5, 32, and 56) were detected in increased amount in PLE conditions developed at low temperatures (Table 3). 

On the other hand, a large number of compounds belonging to the monomers and derivatives group appeared in PLE extracts. With this technique, the behavior of this sub-class was similar to that of gallic acid and derivatives. Indeed, it was observed that the lower the temperature, the more abundant the compounds. The differences between some isomers, which were found at higher temperatures, may be explained by the presence of the monomer subunit, since there was a different extraction response of (+)-catechin (peak 23). This compound showed a similar extraction trend at all temperatures, with increased recovery in the presence of a high percentage of ethanol. On the other hand, (−)-epicatechin (peak 34), which was more abundant, reached high abundance values at low temperatures (Figure 2F). With regard to the SLE technique, most of the compounds were detected with high intensity when the water percentage was higher than the ethanol percentage, except for minor isomers ((epi)catechin gallate isomers, peaks 45 and 48). This could be due to the fact that (+)-catechin was only observed at high percentages of ethanol, whereas there were no significant differences in its recovery at high percentages of water. Concerning the SFE technology (Figure 2E), it is worth stressing that almost no compound was extracted in the SFE-A condition or with very low content. SFE-B was the best condition for obtaining monomers and gallate derivatives. Concerning the (epi)catechin gallate isomer (peak 45), this derivative was extracted by the three techniques, in large amount when using advanced extraction systems (SFE and PLE). Moreover, the SFE-B condition provided the best extraction parameters for obtaining different gallate derivatives, which are compounds with reported biological properties [33,34,35,36]. Nevertheless, the results also indicated that compounds as galloyl-flavan-3-ols showed an increase in the extraction recovery linked to the increase of the ethanol levels applied in the three technologies.

Dimers from the flavan-3-ols and derivatives group represented the largest number of extracted compounds with the three strategies (Figure 2G–I). In SLE, the best conditions were those that included a high proportion of water, except for peaks 9, 21, 25, 37, 38, 42, 47, 50, and 51 that reached the highest intensities when the solvent proportion was 50:50 H_2_O/ethanol (*v*:*v*). Within this chemical group, the most intense peak was an isomer of procyanidin B dimer (peak 47). This compound reached the maximum abundance at 75% of water. Peak 47 was also found at high intensity in PLE experiments developed at high temperature, reaching the highest level in PLE-H. On the contrary, most of the compounds obtained with this methodology showed the same behavior as the monomers and derivatives group, showing the highest recovery when the temperature was low. This could be due to the presence of galloyl groups in their structures. 

Concerning SFE, SFE-B was the best condition to obtain flavan-3-ols and derivatives. However, the procyanidin B dimer was only detected in SFE-C (Figure 2H). This condition differs from SFE-B in that the sample does not contain ethanol. Moreover, any compound in this class could be extracted with SFE-A.

Concerning the flavonoids group, which are non-derivatives of flavan-3-ols (Figure 3A–C), the amounts obtained with the three technologies were generally quite low. PLE provided the extraction conditions that led to better results in terms of number of compounds and abundance. In addition, most compounds belonging to flavonol subclass were extracted at the highest temperature, except for compounds corresponding to peaks 36, 53, 55, and 64. These peaks were characterized as chemical compounds with a glucose unit in their structures. In fact, these four compounds also appeared when using SLE with similar intensity, but aglycones were not extracted by this conventional extraction method at room temperature. In the case of SFE, only two glycosylated flavonoids were detected in condition 2, and a flavanone in SFE-B and 3.

The last category includes other phenolic compounds with different chemical structures, sugars, and others. Comparing the number of compounds obtained with the three techniques, PLE was the best option for the extraction of the compounds in this group (Figure 3F), although the abundance of peaks 1, 2, and 3 was higher when using SLE, in particular in high water percentage conditions (Figure 3D). On the other hand, compounds such as ellagic acid (peak 67) appeared with major intensity when the percentage of ethanol was about 75% using all methodologies, being SFE-B significantly better than the other conditions.

## 3. Materials and Methods

### 3.1. Chemicals and Reagents

All chemicals were of analytical or MS grade and used as received. For extraction, ethanol, sea sand, and glass wood were purchased from Fisher Scientist (Leicestershire, UK). Carbon dioxide was supplied by Carburos Metalicos Grupo Air Products (Carburos Metálicos, Cornellá de Llobregat, Barcelona, Spain). Acetic acid and methanol (MS grade) for HPLC were purchased from Fluka (Sigma-Aldrich, Steinheim, Germany) and Lab-Scan (Gliwice, Sowinskiego, Poland), respectively. Luteolin (≥ 98%) was purchased from Fluka (Sigma-Aldrich, Steinheim, Germany). Double-deionized water with conductivity lower than 18.2 MΩ was obtained with a Milli-Q system from Millipore (Bedford, MA, USA). 

### 3.2. Instrumentation

Supercritical carbon dioxide extraction was carried out with a Waters Prep Supercritical Fluid Extraction system (SFE-100 Waters®, TharSFC, Thar Technologies, Inc., Pittsburgh, PA, USA) equipped with CO_2_ and co-solvent pumps (models P-50), an automated back pressure regulator, low- and high-pressure heating exchangers, a pressurized extraction vessel, and pressurized collection vessels. The SFE system was connected to an Accel 500 LC chiller by Thermo Scientific (TharSFC, Thar Technologies, Inc., Pittsburgh, PA, USA)

Accelerated solvent extraction (ASE) was performed in a Dionex^TM^ ASE 350 extractor (Dionex Corp., Sunnyvale, CA, USA) using a stainless-steel cell of 34 mL volume, and 200 mL vials for extracts collection.

LC analysis of *S. birrea* extracts was performed with an Agilent 1200 series rapid-resolution LC system (Agilent Technologies, Palo Alto, CA, USA) equipped with a binary pump, an autosampler, and a diode array detector (DAD). The HPLC system was coupled to a TOF mass spectrometer (Bruker Daltonics, Bremen, Germany) equipped with an ESI interface (model G1607 from Agilent Technologies, Palo Alto, CA, USA). Separation was carried out with a Zorbax Eclipse Plus C18 (1.8 μm, 150 × 4.6 mm) (Agilent Technologies, Palo Alto, CA, USA).

### 3.3. Sample Preparation

*S. birrea* stem barks were provided by Herbafor S.L. (Murcia, Spain). Stem barks were air-dried and then grounded into uniform powder using an Ultra Centrifugal Mill ZM 200 (Retsch GmbH, Haan, Germany). The resulting *S. birrea* stem bark powder was kept in darkness until used.

### 3.4. Extraction Methods and Conditions

#### 3.4.1. Conventional Solid–Liquid Extraction (SLE)

Phytochemical extraction from *S. birrea* stem bark powder was performed using five different proportions of H_2_O/EtOH (SLE-A, 100:0; SLE-B, 75:25; SLE-C, 50:50; SLE-D, 25:75; and SLE-E, 0:100 (*v*/*v*)). To determine the best SLE condition, 6 g of sample was shaken in dark and at room temperature, for 60 min with 30 mL of the different H_2_O/EtOH mixtures described above. After that, the samples were centrifuged at 13,000 rpm for 10 min in a centrifuge (Sorvall ST 16 R, Thermo Scientific, Leicestershire, UK), and the supernatants were collected and filtered through a 0.45 μm filter. The solvent was evaporated at 35 °C under vacuum in a Savant™ SpeedVac Concentrator SC250 EXP (Thermo Scientific, Sunnyvale, CA, USA). Each procedure was carried out in triplicate. The dried extracts were reconstituted in the extraction solvent up to a concentration of 5 mg/L and filtered through a 0.2 μm nylon syringe filters.

#### 3.4.2. Supercritical Fluid Extraction (SFE)

SFE experiments were performed according to Ghoreishi and Heidari (2012) [37] with some modifications. Briefly, the extractions were carried out in a 100 mL extraction column fitted with glass wool at the inlet and outlet. This column was charged with 15 g of plant powder (SFE-C) plus 10 mL of ethanol (SFE-A and SFE-B) and mixed with sea sand in a ratio of 1:1. All SFE extractions were performed at 50°C and 200 bars in a dynamic mode with a total flow rate of 23 g/min of different solvent combinations (SFE-A, CO_2_; SFE-B and SFE-C, CO_2_ plus ethanol at 15%). All extractions were done in triplicate. The obtained extracts were evaporated and stored at −20 °C until HPLC analysis. For further analysis, the dried extracts were reconstituted in the extraction solvent up to a concentration of 5 mg/L and filtered through a 0.2 μm nylon syringe filters.

#### 3.4.3. Pressurized Liquid Extraction (PLE)

PLE experiments were performed in a static mode with combinations of different proportions of solvent composition (H_2_O/EtOH from 0% to 100%, *v*/*v*) and temperatures in the range from 40 to 200 °C (see Table 3). Prior to use, extraction solvents were degassed for 10 min by using an ultrasonic bath. All extractions were done using 6 g of plant powder previously mixed with sea sand in a ratio of 1:2 and loaded onto 34 mL stainless-steel extraction vessels. In order to prevent clogging of the metal frits, cellulose filters were placed at each end of the extraction vessel, and two portions of sand (5 g) were placed between sample and cellulose filters. The extraction conditions described above were applied, and the extracts were collected in vials and immediately cooled in ice to room temperature. After that, the extracts were centrifuged at 13,000 rpm for 10 min in a centrifuge (Sorvall ST 16 R, Thermo Scientific, Leicestershire, UK). The extracts were dried at 35 °C under vacuum in a Savant™ SpeedVac Concentrator SC250 EXP (Thermo Scientific, Sunnyvale, CA, USA) and stored at −20 °C until HPLC analysis. All extractions were done in triplicate. For further analysis, the dried extracts were reconstituted in the extraction solvent up to a concentration of 5 mg/L and filtered through a 0.2 μm nylon syringe filters.

### 3.5. HPLC–ESI–TOF–MS Analysis

To carry out the chemical characterization of phenolic extracts, HPLC–ESI–TOF–MS analysis was applied following a previously described method by Cádiz-Gurrea et al., with some modifications [38]. The injection volume was 20 μL, and the chromatographic separation was carried out at room temperature. The mobile phase used was water, with 0.5% acetic acid as eluent A and methanol as eluent B. The total run time was 57 min using a previously reported multistep linear gradient [38]. The flow rate was 0.3 mL/min. The HPLC system was coupled to a TOF mass spectrometer equipped with an ESI interface operating in negative-ion mode using a capillary voltage of +4.5 kV. The other optimum values of the source parameters were: drying gas temperature, 210 °C; drying gas flow, 9 L/min; nebulizing gas pressure, 2.3 bar. The detection was performed considering a mass range of 50–1200 *m*/*z*.

External mass spectrometer calibration was performed with sodium acetate clusters (5 mM sodium hydroxide in water/2-propanol 1/1 (*v*/*v*), with 0.2% of acetic) in quadratic high-precision calibration (HPC) regression mode. The calibration solution was injected at the beginning of the run, and all the spectra were calibrated prior to polar compounds identification. The detection was performed considering a mass range of 50–1200 *m*/*z*. The optimum values of the source and transfer parameters were determined for a good sensitivity and reasonable resolution within the mass range described above [38].

### 3.6. Statistical Analysis

Origin (Version Origin Pro 8 SR0, Northampton, MA, USA) was employed to perform one-way analysis of variance (ANOVA) at a 95% confidence level (*p* ≤ 0.05) in order to analyze statistically significant differences among the total phenolic content of extracts obtained under different extraction conditions (Table S1).

## 4. Conclusions

In the present work, a comprehensive characterization by HPLC–ESI–TOF–MS provided a total of 71 compounds, a significant large percentage of which were in a galloyl form, distributed in five major categories. Notably, two gallic acid derivatives were described for the first time in *S. birrea*. Moreover, the main goal of this study was to compare different extraction approaches such as conventional (SLE) and non-conventional or green techniques (SFE and PLE) using generally recognized as safe (GRAS) solvents. In terms of yield, PLE was found to be the best option, since it provided the highest yields when the temperature and the percentage of ethanol were set over 176 °C and 50 % (ethanol/water). 

Different extraction processes lead to the obtainment of different phenolic compounds in relation to their chemical structures. For this reason, the different extraction technologies were evaluated in order to determine the compositional variations of *S. birrea* bark. The largest number of compounds were extracted with PLE, generally when the temperature achieved low values, in particular, all flavonoid aglycones, but not gallic acid and minor (epi)catechin derivatives. In addition, the high percentage of water in the SLE methodology provided a major number of gallic acid derivatives and flavonoids, except for some (epi)catequin derivatives that were obtained when using about 50% of water/ethanol. In the end, SFE-B did prove effective as a way of extracting proanthocyanidins with galloyl residues, probably because of the presence of ethanol as a co-solvent and in the sample.

In the light of our findings, we may conclude that green extraction methods as PLE represent a powerful tool to obtain bioactive compounds from *S. birrea* bark such as galloyl-procyanidins, which can be used as antidiabetic supplements or food ingredients, promoting the valorization of this crop.

## Figures and Tables

**Table 1:** Identified compounds in the *S. birrea* extracts by HPLC–ESI–TOF–MS.

Peak	Proposed Compound	RT	*m*/*z* Calc.	*m*/*z* Meas.	Err [ppm]	mSigma	Mol. Formula	SLE	SFE	PLE
1	Quinic acid	5.6	191.0561	191.0565	2.1	1.6	C_7_H_12_O_6_	A–E	A–C	A *–I
2	Sucrose	7.2	341.1089	341.1095	1.6	0.8	C_12_H_22_O_11_	A–C	nd	A *,B,D–I
3	D-Raffinose	9.9	503.1618	503.1607	2.1	2.7	C_18_H_32_O_16_	A, B	nd	A *–H
4	Galloyl glucose isomer 1	12.4	331.0671	331.0678	2.1	1.5	C_13_H_16_O_10_	A–C	nd	A *–I
5	Galloyl glucose isomer 2	13.1	331.0671	331.0677	1.9	12.6	C_13_H_16_O_10_	A,B	nd	A *,B,G,I
6	Gallo(epi)catechin dimer	13.4	609.1250	609.1228	3.6	3.9	C_30_H_26_O_14_	A *, B	nd	nd
7	Gallic acid	13.7	169.0142	169.0149	3.7	1.8	C_7_H_6_O_5_	A–E	A–C	A *–I
8	UK1	14.4	411.0259	411.0239	−5.0	37.5	C_17_H_8_N_4_O_9_	A *–C	nd	A, B, I
9	Bis(epi)gallocatechin monogallate 1	14.8	761.1359	761.1359	0.1	5.3	C_37_H_30_O_18_	C *, D	nd	nd
10	(Epi)gallocatechin isomer 1	14.8	305.0667	305.0670	1.0	0.9	C_15_H_14_O_7_	A, B	nd	A *–I
11	Bis(epi)gallocatechin monogallate 2	15.2	761.1359	761.1365	0.7	4.6	C_37_H_30_O_18_	A–C, E	nd	A *–I
12	(Epi)gallocatechin gallate (epi)catechin isomer 1	15.5	745.1410	745.1414	−0.5	8.7	C_37_H_30_O_17_	A *–C	nd	nd
13	Protocatechuic acid	15.7	153.0193	153.0192	1.0	5.9	C_7_H_6_O_4_	nd	A, C	G *–I
14	Procyanidin B dimer isomer 1	15.9	577.1351	577.1335	2.8	2.6	C_3_0H_26_O_12_	A *–C	nd	A–F
15	(Epi)catechin-(epi)gallocatechin	16.5	593.1301	593.1311	−1.8	2.7	C_30_H_26_O_13_	A *, B	nd	nd
16	(Epi)gallocatechin gallate (epi)catechin isomer 2	16.7	745.1410	745.1402	1.2	6.8	C_37_H_30_O_17_	A *–C	nd	nd
17	Bis(epi)gallocatechin digallate	16.8	913.1469	913.1493	2.6	2.7	C_44_H_34_O_22_	A-E	nd	A *–I
18	(Epi)gallocatechin gallate (epi)catechin isomer 3	17.4	745.1410	745.1402	1.1	2.7	C_37_H_30_O_17_	A-–E	B, C	A *–G, I
19	(Epi)gallocatechin gallate isomer 1	17.8	457.0776	457.0769	1.7	1.7	C_22_H_18_O_11_	nd	nd	E *–G
20	(Epi)gallocatechin gallate (epi)catechin isomer 4	18.4	745.1410	745.1418	1.0	0.7	C_37_H_30_O_17_	A, E	B	A *–G, I
21	(Epi)gallocatechin gallate (epi)catechin isomer 5	18.6	745.1410	745.1414	0.5	3.7	C_37_H_30_O_17_	C	nd	A *–G
22	(Epi)gallocatechin isomer 2	18.7	305.0667	305.0669	0.8	1.7	C_15_H_14_O_7_	A*–C	nd	nd
23	Catechin	19.4	289.0718	289.0725	2.7	3.2	C_15_H_14_O_6_	A–E	A–C	A*–I
24	(Epi)gallocatechin gallate (epi)catechin gallate isomer 1	19.5	897.1520	897.1543	2.6	7.2	C_44_H_34_O_21_	A–E	nd	A *, B,D–I
25	(Epi)catechin gallate (epi)catechin isomer 1	20	729.1461	729.1471	−1.0	9.8	C_37_H_30_O_16_	B, C	nd	A *, B,D–I
26	(Epi)catechin gallate (epi)catechin isomer 2	20.5	729.1461	729.1476	2.0	3.1	C_37_H_30_O_16_	A–E	B, C	A *–I
27	Protocatechuic acid aldehide	20.5	137.0244	137.0245	−0.5	4.2	C_7_H_6_O_3_	nd	A *	G–I
28	Dimethoxy-hydroxyphenyl-*O*-galloyl-glucopyranoside	20.8	483.1144	483.1134	2.2	4.1	C_21_H_24_O_13_	A *–C	nd	nd
29	(Epi)gallocatechin gallate isomer 2	21.9	457.0776	457.0783	1.5	5.7	C_22_H_18_O_11_	A–E	A–C	A *–I
30	(Epi)catechin gallate (epi)catechin gallate isomer 1	22.2	881.1571	881.1593	−2.6	3.4	C_44_H_33_O_20_	A–E	B, C	A *–G, I
31	(Epi)catechin gallate (epi)catechin gallate isomer 2	23.1	881.1571	881.1586	−1.7	11.8	C_44_H_33_O_20_	A–E	B, C	A *–I
32	Hydroxy-methoxyphenyl-*O*-galloyl-glucopyranoside	23.5	453.1038	453.1042	0.8	2.3	C_20_H_22_O_12_	A–C	nd	A *–G
33	UK2 isomer 1	23.6	439.0671	439.0668	0.5	7.6	C_22_H_16_O_10_	nd	nd	G *–I
34	Epicatechin	24.5	289.0718	289.0722	1.7	2.0	C_15_H_14_O_6_	A–D	A–C	A *–I
35	(Epi)gallocatechin gallate isomer 3	24.7	457.0776	457.0779	0.6	6.0	C_22_H_18_O_11_	C, D	nd	A *, B, E–I
36	Eriodictyol-*O*-glucoside	24.9	449.1089	449.1069	4.6	2.3	C_21_H_22_O_11_	A *–D	nd	A–D
37	(Epi)catechin gallate (epi)catechin gallate isomer 3	25.5	881.1571	881.1586	−1.7	11.8	C_44_H_34_O_20_	A–C	B, C	A *–G, I
38	(Epi)gallocatechin gallate (epi)catechin isomer 6	25.9	745.1410	745.1416	0.8	6.9	C_37_H_30_O_17_	A–D	nd	A *–E, G
39	Galloyl glucosyl dihydroxy methoxyacetophenone	26.2	495.1144	495.1131	2.6	20.4	C_22_H_24_O_13_	A *–C	nd	nd
40	UK2 isomer 2	26.5	439.0671	439.0664	1.5	43.6	C_22_H_16_O_10_	nd	nd	F *–I
41	(Epi)gallocatechin-(epi)catechin-gallate	26.6	743.1254	743.1282	−2.8	50.5	C_37_H_28_O_17_	A *–C	nd	nd
42	(Epi)gallocatechin gallate (epi)catechin gallate isomer 2	26.7	897.1520	897.1516	0.4	6.1	C_44_H_34_O_21_	A *–C	nd	A–C
43	Lyoniside	27.5	551.2134	551.2136	0.4	4.2	C_27_H_36_O_12_	A *–C	nd	A–G
44	(Epi)catechin gallate isomer 1	27.9	441.0827	441.0836	2.1	2.3	C_22_H_18_O_10_	nd	A *	G–I
45	(Epi)catechin gallate isomer 2	28.2	441.0827	441.0836	2.0	2.0	C_22_H_18_O_10_	A–E	B, C	A *–I
46	(Epi)catechin-3-*O*-glucoside-gallate	28.9	603.1355	603.1355	0.1	15.1	C_28_H_28_O_15_	A *–C, E	nd	A–G
47	Procyanidin B dimer isomer 2	29.6	577.1351	577.1346	0.9	12.1	C_30_H_26_O_12_	A–D	C	A *–E, G–I
48	(Epi)catechin gallate isomer 3	30.1	441.0827	441.0814	2.9	2.4	C_22_H_18_O_10_	A–E	B, C	A *–I
49	Dihydromyricetin isomer 1	31	319.0459	319.0465	1.7	0.3	C_15_H_12_O_8_	nd	nd	G *–I
50	(Epi)catechin gallate (epi)catechin gallate isomer 4	31.8	881.1571	881.1602	−3.6	5.0	C_44_H_34_O_20_	A–E	B, C	A *–I
51	(Epi)catechin gallate (epi)catechin isomer 3	32.1	729.1461	729.1488	3.8	2.8	C_37_H_30_O_16_	A–E	B, C	A *–I
52	(Epi)afzelechin gallate	33	425.0878	425.0892	3.2	9.5	C_22_H_18_O_9_	A–E	B, C	A *–G
53	Myricetin glucoside	33.5	479.0831	479.0849	−3.8	14.0	C_21_H_20_O_13_	A *–E	B	A–C, F
54	Jaceidin triacetate	33.6	485.1089	485.1093	0.8	8.3	C_24_H_22_O_11_	nd	nd	D *, E, G–I
55	Phloretin-di-C-glucoside	34.6	597.1825	597.1807	3.0	13.1	C_27_H_34_O_15_	A *–E	nd	A, B
56	Trihydroxystilbene glucosyl-*O*-gallate	34.7	541.1351	541.1362	−1.9	14.9	C_27_H_26_O_12_	A *–E	B	A, B, D–F
57	Dihydromyricetin isomer 2	35.6	319.0459	319.0465	1.7	0.3	C_15_H_12_O_8_	nd	nd	E, G *–I
58	UK3	35.7	439.1093	439.1073	4.5	36.7	C_16_H_24_O_14_	A *–C, E	B	A, B
59	Homaloside D	36.4	543.1508	543.1529	−3.8	19.2	C_27_H_28_O_12_	A *–C	nd	A–C
60	Phloretin-C-glucoside (nothofagin)	36.4	435.1297	435.1281	3.6	8.8	C_21_H_24_O_10_	C *, E	B	nd
61	Rhamnetin	36.6	315.0510	315.0510	−0.1	46.1	C_16_H_12_O_7_	nd	nd	G *–I
62	Dihydroquercetin	37.6	303.0510	303.0514	1.2	17.9	C_15_H_12_O_7_	nd	nd	D *–I
63	Pentamethoxystilbene isomer 1	37.7	329.1394	329.1402	2.3	6.8	C_19_H_22_O_5_	B *, C, E	A–C	A, B, E
64	Quercetin glucoside	37.9	463.0882	463.0883	−0.3	0.7	C_21_H_20_O_12_	C *, E	nd	A, B
65	Syringic aldehyde	38.7	181.0506	181.0503	1.7	0.8	C_9_H_10_O_4_	nd	nd	G *–I
66	Pentamethoxystilbene isomer 2	38.9	329.1394	329.1387	2.4	5.5	C_19_H_22_O_5_	A *–E	A-C	A–F
67	Ellagic acid	40.2	300.9990	301.0004	4.6	15.7	C_14_H_6_O_8_	A–E	B, C	A *–I
68	Naringenin	40.8	271.0612	271.0609	1.3	7.8	C_15_H_12_O_5_	nd	nd	F, G *, I
69	Taxifolin	41.6	303.0510	303.0519	2.8	0.9	C_15_H_12_O_7_	nd	nd	E, G *–I
70	Nonanedioic acid (azelaic acid)	42.3	187.0981	187.0976	−2.8	5.1	C_9_H_16_O_4_	nd	A *–C	nd
71	Flavanone	47.2	271.0612	271.0622	−3.5	8.3	C_15_H_12_O_5_	nd	B *, C	nd

* RT: retention time, calc. and meas. *m*/*z*, error, and σ values are referred to this extract; nd, Non-detected; UK, unknown; SLE-A, 100:0; SLE-B, 75:25; SLE-C, 50:50; SLE-D, 25:75; and SLE-E, 0:100 (H_2_O, EtOH; *v*/*v*). SFE-A: 15 g + 10 mL EtOH, CO_2_; SFE-B: 15 g + 10 mL EtOH, CO_2_ plus ethanol at 15%; SFE-C: 15 g, CO_2_ plus ethanol at 15%. PLE-A, 40 °C, 50:50; PLE-B, 63 °C, 85:15; PLE-C, 63 °C, 15:85; PLE-D, 120 °C, 100:0; PLE-E, 120 °C, 50:50; PLE-F, 120 °C, 0:100; PLE-G, 176 °C, 85:15; PLE-H, 176 °C, 15:85; PLE-I, 200 °C, 50:50; (H_2_O, EtOH; *v*/*v*).

**Table 2:** Values of extraction yield (%) obtained in the different conditions for each extraction methodology.

SLE					SFE			PLE								
A	B	C	D	E	A	B	C	A	B	C	D	E	F	G	H	I
6.2 ± 0.2	12 ± 1	12.1 ± 0.2	9.3 ± 0.5	7 ± 1	0.6 ± 0.2	2.2 ± 0.4	0.6 ± 0.1	23.0 ± 0.3	20 ± 3	25 ± 1	20 ± 1	29 ± 1	21 ± 2	31 ± 3	21 ± 2	42 ± 1

SLE-A, 100:0; SLE-B, 75:25; SLE-C, 50:50; SLE-D, 25:75; and SLE-E, 0:100 (H_2_O, EtOH; *v*/*v*). SFE-A: 15 g +10 mL EtOH, CO_2_; SFE-B: 15 g + 10 mL EtOH, CO_2_ plus ethanol at 15%; SFE-C: 15 g, CO_2_ plus ethanol at 15%. PLE-A, 40 °C, 50:50; PLE-B, 63 °C, 85:15; PLE-C, 63 °C, 15:85; PLE-D, 120 °C, 100:0; PLE-E, 120 °C, 50:50; PLE-F, 120 °C, 0:100; PLE-G, 176 °C, 85:15; PLE-H, 176 °C, 15:85; PLE-I, 200 °C, 50:50; (H_2_O, EtOH; *v*/*v*)

**Table 3:** Extraction parameters of PLE.

Conditions	P (psi)	T (min)	T (°C)	EtOH (%)	H_2_O (%)	Dielectric Constant
A	1500	20	40	50	50	48.02
B			63	85	15	31.02
C			63	15	85	59.09
D			120	100	0	19.00
E			120	50	50	34.71
F			120	0	100	50.41
G			176	85	15	21.55
H			176	15	85	33.43
I			200	50	50	26.00

## Supplementary Materials

Supplementary materials can be found online. Table S1. Statistical data (ANOVA) of all compounds with the different extraction methodologies; Table S2. Means of comparison of the ratio of the analyte peak area to the internal standard peak area for compounds extracted by SLE; Table S3. Means of comparison of the ratio of the analyte peak area to the internal standard peak area for compounds extracted by SFE; Table S4. Means of comparison of the ratio of the analyte peak area to the internal standard peak area for compounds extracted by PLE; Table S5. Statistical data (ANOVA) of the best extraction condition for compounds extracted by all methodologies.
