# Supplementary material for: Enhancing the Yield of Bioactive Compounds from Sclerocarya birrea Bark by Green Extraction Approaches
Source: Molecules. 2019 Mar 9;24(5):966. doi: 10.3390/molecules24050966 (PMC6429519; doi:10.3390/molecules24050966)
Supplement: Supplementary file 1 [file molecules-24-00966-s001.zip › Supplementary Material-molecules.docx]

Supplementary Material

Enhancing obtainment of antidiabetic compounds from *Sclerocarya birrea* bark by green extraction approaches

María de la Luz Cádiz-Gurrea ^1,2,*^, Jesús Lozano-Sánchez ^1,2^, Álvaro Fernández-Ochoa ^1,2^ and Antonio Segura-Carretero ^1,2,*^

^1^ Department of Analytical Chemistry, University of Granada, C/ Fuentenueva s/n, 18071 Granada, Spain; [mluzcadiz@ugr.es](mailto:mluzcadiz@ugr.es), [jesusls@ugr.es](mailto:jesusls@ugr.es), [alvaroferochoa@ugr.es](mailto:alvaroferochoa@ugr.es), [ansegura@ugr.es](mailto:ansegura@ugr.es)

^2^ Research and Development of Functional Food Centre (CIDAF), PTS Granada, Avda. Del Conocimiento s/n., Edificio BioRegion, 18016 Granada, Spain; e-mail@e-mail.com

***** Correspondence: ansegura@ugr.es; Tel.: +34-958-637-206

| **Table S2. Means of comparison of the ratio of analyte peak area to internal standard peak area for compounds extracted by SLE.** | | | | | | | | | | |
| --- | --- | --- | --- | --- | --- | --- | --- | --- | --- | --- |
| **Peak** | **SLE-A** | **SD** | **SLE-B** | **SD** | **SLE-C** | **SD** | **SLE-D** | **SD** | **SLE-E** | **SD** |
| **1** | 7.772 | 0.629 | 4.504 | 0.176 | 3.724 | 0.029 | 2.689 | 0.041 | 1.781 | 0.023 |
| **2** | 0.485 | 0.046 | 0.083 | 0.003 | 0.323 | 0.019 |  |  |  |  |
| **3** | 0.612 | 0.013 | 0.182 | 0.005 |  |  |  |  |  |  |
| **4** | 0.291 | 0.124 | 0.060 | 0.002 | 0.074 | 0.002 |  |  |  |  |
| **5** | 0.089 | 0.003 | 0.032 | 0.001 |  |  |  |  |  |  |
| **6** | 0.058 | 0.007 | 0.105 | 0.009 |  |  |  |  |  |  |
| **7** | 1.078 | 0.093 | 1.140 | 0.016 | 0.770 | 0.016 | 0.469 | 0.053 | 0.281 | 0.019 |
| **8** | 0.364 | 0.032 | 0.100 | 0.014 | 0.051 | 0.004 |  |  |  |  |
| **9** |  |  |  |  | 0.060 | 0.004 | 0.085 | 0.003 |  |  |
| **10** | 0.102 | 0.009 | 0.132 | 0.003 |  |  |  |  |  |  |
| **11** | 0.347 | 0.037 | 0.297 | 0.017 | 0.186 | 0.008 |  |  | 0.072 | 0.002 |
| **12** | 0.054 | 0.008 | 0.083 | 0.005 | 0.018 | 0.002 |  |  |  |  |
| **14** | 0.099 | 0.014 | 0.079 | 0.006 | 0.036 | 0.003 |  |  |  |  |
| **15** | 0.108 | 0.011 | 0.200 | 0.007 |  |  |  |  |  |  |
| **16** | 0.092 | 0.010 | 0.087 | 0.002 | 0.146 | 0.008 |  |  |  |  |
| **17** | 0.174 | 0.008 | 0.085 | 0.006 | 0.151 | 0.003 | 0.073 | 0.001 | 0.046 | 0.001 |
| **18** | 0.494 | 0.039 | 0.442 | 0.010 | 0.358 | 0.012 | 0.148 | 0.006 | 0.119 | 0.003 |
| **20** | 0.342 | 0.044 | 0.280 | 0.007 |  |  |  |  | 0.077 | 0.001 |
| **21** |  |  |  |  | 0.181 | 0.006 |  |  |  |  |
| **22** | 0.204 | 0.024 | 0.301 | 0.011 | 0.075 | 0.003 |  |  |  |  |
| **23** | 0.151 | 0.014 | 0.146 | 0.003 | 0.142 | 0.001 | 0.067 | 0.002 | 0.036 | 0.001 |
| **24** | 0.182 | 0.010 | 0.293 | 0.014 | 0.250 | 0.005 | 0.115 | 0.009 | 0.080 | 0.002 |
| **25** |  |  | 0.019 | 0.001 | 0.276 | 0.012 |  |  |  |  |
| **26** | 0.743 | 0.075 | 0.668 | 0.019 | 0.461 | 0.018 | 0.287 | 0.009 | 0.268 | 0.007 |
| **28** | 0.063 | 0.005 | 0.045 | 0.001 | 0.029 | 0.003 |  |  |  |  |
| **29** | 0.448 | 0.028 | 0.426 | 0.012 | 0.561 | 0.009 | 0.453 | 0.012 | 0.301 | 0.013 |
| **30** | co0.244 | 0.021 | 0.114 | 0.010 | 0.233 | 0.019 | 0.170 | 0.005 | 0.142 | 0.008 |
| **Table S2. Cont.** | | | | | | | | | | |
| **31** | 0.054 | 0.007 | 0.051 | 0.003 | 0.061 | 0.003 | 0.029 | 0.003 | 0.028 | 0.003 |
| **32** | 0.044 | 0.005 | 0.030 | 0.001 | 0.030 | 0.004 |  |  |  |  |
| **34** | 0.495 | 0.077 | 0.769 | 0.009 | 0.283 | 0.005 | 0.144 | 0.002 |  |  |
| **35** |  |  |  |  | 0.095 | 0.002 | 0.060 | 0.001 |  |  |
| **36** | 0.103 | 0.015 | 0.061 | 0.002 | 0.054 | 0.001 | 0.028 | 0.006 |  |  |
| **37** | 0.018 | 0.002 | 0.014 | 0.001 | 0.024 | 0.002 |  |  |  |  |
| **38** | 0.037 | 0.006 | 0.076 | 0.003 | 0.047 | 0.002 | 0.022 | 0.008 |  |  |
| **39** | 0.030 | 0.004 | 0.018 | 0.001 | 0.018 | 0.002 |  |  |  |  |
| **41** | 0.040 | 0.005 | 0.026 | 0.001 | 0.024 | 0.002 |  |  |  |  |
| **42** | 0.029 | 0.002 | 0.021 | 0.001 | 0.045 | 0.003 |  |  |  |  |
| **43** | 0.036 | 0.001 | 0.028 | 0.001 | 0.025 | 0.001 |  |  |  |  |
| **45** | 1.297 | 0.102 | 1.077 | 0.008 | 1.415 | 0.020 | 1.483 | 0.036 | 1.251 | 0.025 |
| **46** | 0.046 | 0.000 | 0.027 | 0.002 | 0.044 | 0.001 |  |  | 0.025 | 0.002 |
| **47** | 0.008 | 0.001 | 3.013 | 0.068 | 1.009 | 0.009 | 0.070 | 0.001 |  |  |
| **48** | 0.063 | 0.005 | 0.054 | 0.001 | 0.176 | 0.004 | 0.083 | 0.012 | 0.081 | 0.003 |
| **50** | 0.090 | 0.001 | 0.062 | 0.005 | 0.150 | 0.001 | 0.069 | 0.007 | 0.075 | 0.001 |
| **51** | 0.188 | 0.012 | 0.272 | 0.010 | 0.197 | 0.007 | 0.112 | 0.002 | 0.108 | 0.002 |
| **52** | 0.018 | 0.000 | 0.015 | 0.000 | 0.036 | 0.001 | 0.034 | 0.000 | 0.022 | 0.001 |
| **53** | 0.028 | 0.001 | 0.024 | 0.002 | 0.028 | 0.001 | 0.021 | 0.003 | 0.015 | 0.002 |
| **55** | 0.026 | 0.002 | 0.020 | 0.001 | 0.022 | 0.001 | 0.019 | 0.002 | 0.012 | 0.001 |
| **56** | 0.021 | 0.001 | 0.021 | 0.001 | 0.035 | 0.002 | 0.026 | 0.005 | 0.013 | 0.001 |
| **58** | 0.102 | 0.008 | 0.079 | 0.000 | 0.079 | 0.001 |  |  | 0.029 | 0.000 |
| **59** | 0.030 | 0.000 | 0.023 | 0.001 | 0.023 | 0.001 |  |  |  |  |
| **60** |  |  |  |  | 0.014 | 0.000 |  |  | 0.007 | 0.000 |
| **63** |  |  | 0.014 | 0.001 | 0.016 | 0.001 |  |  | 0.010 | 0.001 |
| **64** |  |  |  |  | 0.015 | 0.001 |  |  | 0.009 | 0.001 |
| **66** | 0.028 | 0.000 | 0.032 | 0.000 | 0.033 | 0.001 | 0.030 | 0.000 | 0.024 | 0.000 |
| **67** | 0.093 | 0.001 | 0.079 | 0.002 | 0.127 | 0.004 | 0.166 | 0.003 | 0.084 | 0.008 |

| **Table S3. Means of comparison of the ratio of analyte peak area to internal standard peak area for compounds extracted by SFE.** | | | | | | |
| --- | --- | --- | --- | --- | --- | --- |
| **Peak** | **SFE-A** | **SD** | **SFE-B** | **SD** | **SFE-C** | **SD** |
| **1** | 0.254 | 0.014 | 2.794 | 0.404 | 0.747 | 0.017 |
| **7** | 1.444 | 0.056 | 1.507 | 0.024 | 0.648 | 0.066 |
| **13** | 0.039 | 0.009 |  |  | 0.033 | 0.002 |
| **18** |  |  | 0.109 | 0.024 | 0.039 | 0.001 |
| **20** |  |  | 0.082 | 0.002 |  |  |
| **23** | 0.018 | 0.002 | 0.381 | 0.055 | 0.107 | 0.004 |
| **26** |  |  | 0.454 | 0.006 | 0.162 | 0.001 |
| **27** | 0.100 | 0.007 |  |  |  |  |
| **29** | 0.019 | 0.002 | 0.835 | 0.051 | 0.234 | 0.010 |
| **Table S3. Cont.** | | | | | | |
| **30** |  |  | 0.238 | 0.009 | 0.089 | 0.002 |
| **31** |  |  | 0.604 | 0.025 | 0.252 | 0.008 |
| **34** | 0.026 | 0.001 | 0.604 | 0.025 | 0.227 | 0.002 |
| **37** |  |  | 0.839 | 0.027 | 0.212 | 0.008 |
| **44** | 0.168 | 0.005 |  |  |  |  |
| **45** |  |  | 2.102 | 0.114 | 1.123 | 0.008 |
| **47** |  |  |  |  | 0.030 | 0.001 |
| **48** |  |  | 0.411 | 0.003 | 0.050 | 0.001 |
| **50** |  |  | 0.109 | 0.003 | 0.022 | 0.000 |
| **51** |  |  | 0.158 | 0.004 | 0.043 | 0.001 |
| **52** |  |  | 0.090 | 0.002 | 0.017 | 0.001 |
| **53** |  |  | 0.034 | 0.003 |  |  |
| **56** |  |  | 0.033 | 0.002 |  |  |
| **58** |  |  | 0.024 | 0.001 |  |  |
| **60** |  |  | 0.028 | 0.001 |  |  |
| **63** | 0.026 | 0.004 | 0.073 | 0.005 | 0.039 | 0.001 |
| **66** | 0.040 | 0.002 | 0.116 | 0.006 | 0.061 | 0.001 |
| **67** |  |  | 0.348 | 0.085 | 0.045 | 0.004 |
| **70** | 0.135 | 0.001 | 0.060 | 0.003 | 0.107 | 0.004 |
| **71** |  |  | 0.028 | 0.002 | 0.026 | 0.001 |

| **Table S4. Means of comparison of the ratio of analyte peak area to internal standard peak area for compounds extracted by PLE.** | | | | | | | | | | | | | | | | | | |
| --- | --- | --- | --- | --- | --- | --- | --- | --- | --- | --- | --- | --- | --- | --- | --- | --- | --- | --- |
| **Peak** | **PLE-A** | **SD** | **PLE-B** | **SD** | **PLE-C** | **SD** | **PLE-D** | **SD** | **PLE-E** | **SD** | **PLE-F** | **SD** | **PLE-G** | **SD** | **PLE-H** | **SD** | **PLE-I** | **SD** |
| **1** | 2.511 | 0.035 | 2.180 | 0.024 | 2.490 | 0.099 | 1.552 | 0.021 | 2.311 | 0.018 | 2.202 | 0.087 | 2.276 | 0.071 | 2.006 | 0.123 | 2.791 | 0.074 |
| **2** | 0.135 | 0.004 | 0.283 | 0.004 |  |  | 0.183 | 0.002 | 0.116 | 0.000 | 0.101 | 0.003 | 0.120 | 0.014 | 0.063 | 0.002 | 0.064 | 0.002 |
| **3** | 0.052 | 0.003 | 0.081 | 0.001 | 0.056 | 0.004 | 0.064 | 0.001 | 0.060 | 0.003 | 0.025 | 0.004 | 0.041 | 0.001 | 0.017 | 0.001 |  |  |
| **4** | 0.191 | 0.004 | 0.171 | 0.003 | 0.098 | 0.010 | 0.093 | 0.004 | 0.081 | 0.002 | 0.033 | 0.000 | 0.060 | 0.004 | 0.033 | 0.003 | 0.031 | 0.003 |
| **5** | 0.035 | 0.000 | 0.028 | 0.001 |  |  |  |  |  |  |  |  | 0.032 | 0.004 |  |  | 0.035 | 0.001 |
| **7** | 0.644 | 0.010 | 0.646 | 0.015 | 0.704 | 0.024 | 0.739 | 0.002 | 0.835 | 0.004 | 1.049 | 0.017 | 1.504 | 0.036 | 0.847 | 0.042 | 1.455 | 0.077 |
| **8** | 0.102 | 0.008 | 0.055 | 0.005 |  |  |  |  |  |  |  |  |  |  |  |  | 0.043 | 0.003 |
| **10** | 0.071 | 0.000 | 0.095 | 0.002 | 0.062 | 0.002 | 0.089 | 0.001 | 0.096 | 0.001 | 0.098 | 0.000 | 0.128 | 0.011 | 0.076 | 0.001 | 0.063 | 0.002 |
| **11** | 0.219 | 0.001 | 0.229 | 0.003 | 0.133 | 0.010 | 0.142 | 0.001 | 0.116 | 0.003 | 0.095 | 0.004 | 0.078 | 0.007 | 0.031 | 0.000 | 0.029 | 0.001 |
| **13** |  |  |  |  |  |  |  |  |  |  |  |  | 0.158 | 0.006 | 0.340 | 0.015 | 0.267 | 0.006 |
| **14** | 0.088 | 0.002 | 0.108 | 0.005 | 0.066 | 0.003 | 0.084 | 0.005 | 0.048 | 0.001 | 0.041 | 0.002 |  |  |  |  |  |  |
| **17** | 0.204 | 0.003 | 0.242 | 0.004 | 0.125 | 0.017 | 0.146 | 0.001 | 0.127 | 0.002 | 0.085 | 0.003 | 0.098 | 0.006 | 0.048 | 0.002 | 0.050 | 0.002 |
| **18** | 0.499 | 0.005 | 0.440 | 0.005 | 0.279 | 0.032 | 0.343 | 0.003 | 0.278 | 0.001 | 0.244 | 0.007 | 0.215 | 0.016 |  |  | 0.101 | 0.003 |
| **19** |  |  |  |  |  |  |  |  | 0.079 | 0.000 | 0.025 | 0.002 | 0.050 | 0.004 |  |  |  |  |
| **20** | 0.356 | 0.005 | 0.300 | 0.004 | 0.201 | 0.015 | 0.252 | 0.001 | 0.161 | 0.001 | 0.131 | 0.005 | 0.144 | 0.010 |  |  | 0.066 | 0.002 |
| **21** | 0.086 | 0.001 | 0.086 | 0.002 | 0.087 | 0.002 | 0.082 | 0.001 | 0.047 | 0.001 | 0.047 | 0.002 | 0.037 | 0.005 |  |  |  |  |
| **23** | 0.171 | 0.001 | 0.234 | 0.002 | 0.211 | 0.006 | 0.250 | 0.003 | 0.200 | 0.002 | 0.194 | 0.007 | 0.235 | 0.005 | 0.169 | 0.008 | 0.139 | 0.004 |
| **24** | 0.258 | 0.001 | 0.245 | 0.007 |  |  | 0.218 | 0.017 | 0.199 | 0.002 | 0.151 | 0.005 | 0.169 | 0.008 | 0.095 | 0.002 | 0.093 | 0.002 |
| **25** | 0.052 | 0.002 | 0.043 | 0.001 |  |  | 0.043 | 0.003 | 0.086 | 0.001 | 0.090 | 0.002 | 0.079 | 0.008 | 0.061 | 0.001 | 0.063 | 0.001 |
| **26** | 0.781 | 0.004 | 0.624 | 0.002 | 0.554 | 0.019 | 0.619 | 0.008 | 0.460 | 0.004 | 0.432 | 0.013 | 0.401 | 0.013 | 0.162 | 0.018 | 0.210 | 0.004 |
| **27** |  |  |  |  |  |  |  |  |  |  |  |  | 0.177 | 0.012 | 0.585 | 0.003 | 0.333 | 0.030 |
| **29** | 0.484 | 0.003 | 0.530 | 0.004 | 0.459 | 0.011 | 0.505 | 0.007 | 0.492 | 0.001 | 0.351 | 0.014 | 0.435 | 0.007 | 0.421 | 0.014 | 0.356 | 0.004 |
| **30** | 0.426 | 0.003 | 0.350 | 0.008 | 0.284 | 0.002 | 0.372 | 0.005 | 0.283 | 0.002 | 0.201 | 0.005 | 0.254 | 0.018 |  |  | 0.132 | 0.006 |
| **31** | 0.102 | 0.002 | 0.086 | 0.006 | 0.070 | 0.001 | 0.076 | 0.003 | 0.088 | 0.003 | 0.099 | 0.006 | 0.100 | 0.014 | 0.068 | 0.001 | 0.070 | 0.001 |
| **32** | 0.030 | 0.001 | 0.042 | 0.001 | 0.046 | 0.003 | 0.022 | 0.001 | 0.021 | 0.002 | 0.024 | 0.002 | 0.019 | 0.000 |  |  |  |  |
| **Table S4. Cont.** | | | | | | | | | | | | | | | | | | |
| **33** |  |  |  |  |  |  |  |  |  |  |  |  | 0.136 | 0.009 | 0.108 | 0.005 | 0.033 | 0.003 |
| **34** | 0.301 | 0.001 | 0.266 | 0.002 | 0.276 | 0.024 | 0.309 | 0.004 | 0.178 | 0.002 | 0.170 | 0.004 | 0.187 | 0.004 | 0.128 | 0.007 | 0.113 | 0.005 |
| **35** | 0.102 | 0.001 | 0.114 | 0.002 |  |  |  |  | 0.224 | 0.005 | 0.252 | 0.014 | 0.389 | 0.009 | 0.320 | 0.012 | 0.279 | 0.007 |
| **36** | 0.096 | 0.000 | 0.069 | 0.000 | 0.059 | 0.014 | 0.062 | 0.002 |  |  |  |  |  |  |  |  |  |  |
| **37** | 0.053 | 0.006 | 0.040 | 0.002 | 0.024 | 0.002 | 0.038 | 0.001 | 0.045 | 0.001 | 0.041 | 0.003 | 0.052 | 0.016 |  |  | 0.042 | 0.001 |
| **38** | 0.054 | 0.000 | 0.045 | 0.000 | 0.024 | 0.001 | 0.038 | 0.003 | 0.019 | 0.001 |  |  | 0.030 | 0.002 |  |  |  |  |
| **40** |  |  |  |  |  |  |  |  |  |  | 0.034 | 0.006 | 0.148 | 0.008 | 0.087 | 0.001 | 0.067 | 0.004 |
| **42** | 0.055 | 0.001 | 0.057 | 0.001 | 0.029 | 0.005 |  |  |  |  |  |  |  |  |  |  |  |  |
| **43** | 0.027 | 0.000 | 0.028 | 0.000 | 0.029 | 0.001 | 0.028 | 0.000 | 0.021 | 0.000 | 0.026 | 0.001 | 0.020 | 0.000 |  |  |  |  |
| **44** |  |  |  |  |  |  |  |  |  |  |  |  | 0.049 | 0.005 | 0.017 | 0.000 | 0.027 | 0.003 |
| **45** | 1.547 | 0.011 | 1.476 | 0.012 | 1.160 | 0.028 | 1.354 | 0.014 | 1.173 | 0.009 | 0.956 | 0.010 | 1.108 | 0.054 | 1.049 | 0.038 | 0.896 | 0.030 |
| **46** | 0.077 | 0.001 | 0.060 | 0.004 | 0.041 | 0.002 | 0.034 | 0.018 | 0.031 | 0.003 | 0.026 | 0.002 | 0.036 | 0.001 |  |  |  |  |
| **47** | 0.192 | 0.005 | 0.009 | 0.000 | 0.277 | 0.015 | 0.074 | 0.001 | 0.115 | 0.000 |  |  | 1.661 | 0.086 | 2.625 | 0.098 | 1.282 | 0.046 |
| **48** | 0.243 | 0.003 | 0.221 | 0.003 | 0.112 | 0.002 | 0.188 | 0.013 | 0.354 | 0.004 | 0.486 | 0.002 | 0.848 | 0.041 | 0.702 | 0.022 | 0.640 | 0.018 |
| **49** |  |  |  |  |  |  |  |  |  |  |  |  | 0.336 | 0.015 | 0.088 | 0.001 | 0.092 | 0.006 |
| **50** | 0.241 | 0.002 | 0.193 | 0.002 | 0.066 | 0.001 | 0.240 | 0.078 | 0.082 | 0.007 | 0.022 | 0.000 | 0.109 | 0.014 | 0.032 | 0.001 | 0.023 | 0.001 |
| **51** | 0.283 | 0.001 | 0.197 | 0.000 | 0.107 | 0.006 | 0.207 | 0.000 | 0.098 | 0.001 | 0.045 | 0.000 | 0.115 | 0.007 | 0.044 | 0.009 | 0.043 | 0.001 |
| **52** | 0.052 | 0.000 | 0.047 | 0.001 | 0.032 | 0.015 | 0.039 | 0.000 | 0.024 | 0.000 | 0.016 | 0.000 | 0.033 | 0.003 |  |  |  |  |
| **53** | 0.031 | 0.001 | 0.036 | 0.002 | 0.017 | 0.002 |  |  |  |  | 0.019 | 0.001 |  |  |  |  |  |  |
| **54** |  |  |  |  |  |  | 0.493 | 0.004 | 0.133 | 0.001 |  |  | 0.252 | 0.019 | 0.085 | 0.001 | 0.028 | 0.001 |
| **55** | 0.029 | 0.001 | 0.023 | 0.001 |  |  |  |  |  |  |  |  |  |  |  |  |  |  |
| **56** | 0.038 | 0.009 | 0.044 | 0.001 |  |  | 0.032 | 0.002 | 0.028 | 0.001 | 0.021 | 0.001 |  |  |  |  |  |  |
| **57** |  |  |  |  |  |  |  |  | 0.027 | 0.001 |  |  | 0.410 | 0.018 | 0.123 | 0.002 | 0.125 | 0.005 |
| **58** | 0.086 | 0.001 | 0.088 | 0.004 |  |  |  |  |  |  |  |  |  |  |  |  |  |  |
| **59** | 0.022 | 0.000 | 0.026 | 0.001 | 0.020 | 0.003 |  |  |  |  |  |  |  |  |  |  |  |  |
| **61** |  |  |  |  |  |  |  |  |  |  |  |  | 0.039 | 0.002 | 0.360 | 0.016 | 0.062 | 0.001 |
| **Table S4. Cont.** | | | | | | | | | | | | | | | | | | |
| **62** |  |  |  |  |  |  | 0.046 | 0.001 | 0.044 | 0.001 | 0.029 | 0.001 | 0.821 | 0.003 | 0.392 | 0.017 | 0.302 | 0.003 |
| **63** | 0.015 | 0.000 | 0.021 | 0.000 |  |  |  |  | 0.014 | 0.000 |  |  |  |  |  |  |  |  |
| **64** | 0.018 | 0.001 | 0.018 | 0.001 |  |  |  |  |  |  |  |  |  |  |  |  |  |  |
| **65** |  |  |  |  |  |  |  |  |  |  |  |  | 0.077 | 0.002 | 0.120 | 0.005 | 0.039 | 0.004 |
| **66** | 0.030 | 0.000 | 0.040 | 0.000 | 0.025 | 0.000 | 0.040 | 0.000 | 0.030 | 0.000 | 0.026 | 0.000 |  |  |  |  |  |  |
| **67** | 0.082 | 0.002 | 0.116 | 0.003 | 0.092 | 0.001 | 0.147 | 0.000 | 0.143 | 0.002 | 0.112 | 0.005 | 0.170 | 0.000 | 0.125 | 0.003 | 0.062 | 0.001 |
| **68** |  |  |  |  |  |  |  |  |  |  | 0.017 | 0.000 | 0.100 | 0.012 |  |  | 0.030 | 0.001 |
| **69** |  |  |  |  |  |  |  |  | 0.027 | 0.001 |  |  | 0.888 | 0.042 | 0.383 | 0.015 | 0.289 | 0.001 |

| **Table S5.** Statistical data (ANOVA) of best extraction condition for compounds which appear in all methodologies. | | | | | | | | | | | | | | | | | | | | | | | |
| --- | --- | --- | --- | --- | --- | --- | --- | --- | --- | --- | --- | --- | --- | --- | --- | --- | --- | --- | --- | --- | --- | --- | --- |
| **Peak** | | 1 | **Peak** | | 7 | **Peak** | | 18 | **Peak** | | 20 | **Peak** | | 23 | **Peak** | | 26 | **Peak** | | 29 | **Peak** | | 30 |
| **m/z** | | 191 | **m/z** | | 169 | **m/z** | | 745 | **m/z** | | 745 | **m/z** | | 289 | **m/z** | | 729 | **m/z** | | 457 | **m/z** | | 881 |
| PLE-G | SFE-B | 0 | PLE-G | SFE-B | 0 | PLE-A | SFE-B | 1 | PLE-A | SFE-B | 1 | PLE-D | SFE-B | 1 | PLE-A | SFE-B | 1 | PLE-B | SFE-B | 0 | PLE-A | SFE-B | 1 |
|  | SLE-A | 1 |  | SLE-B | 1 |  | SLE-A | 0 |  | SLE-A | 0 |  | SLE-A | 1 |  | SLE-A | 0 |  | SLE-C | 1 |  | SLE-A | 1 |
| SFE-B | PLE-G | 0 | SFE-B | PLE-G | 0 | SFE-B | PLE-A | 1 | SFE-B | PLE-A | 1 | SFE-B | PLE-D | 1 | SFE-B | PLE-A | 1 | SFE-B | PLE-B | 0 | SFE-B | PLE-A | 1 |
|  | SLE-A | 1 |  | SLE-B | 1 |  | SLE-A | 1 |  | SLE-A | 1 |  | SLE-A | 1 |  | SLE-A | 1 |  | SLE-C | 1 |  | SLE-A | 0 |
| SLE-A | PLE-G | 1 | SLE-B | PLE-G | 1 | SLE-A | PLE-A | 0 | SLE-A | PLE-A | 0 | SLE-A | PLE-D | 1 | SLE-A | PLE-A | 0 | SLE-C | PLE-B | 1 | SLE-A | PLE-A | 1 |
|  | SFE-B | 1 |  | SFE-B | 1 |  | SFE-B | 1 |  | SFE-B | 1 |  | SFE-B | 1 |  | SFE-B | 1 |  | SFE-B | 1 |  | SFE-B | 0 |
| **Peak** | | 31 | **Peak** | | 34 | **Peak** | | 37 | **Peak** | | 44 | **Peak** | | 45 | **Peak** | | 47 | **Peak** | | 48 | **Peak** | | 50 |
| **m/z** | | 881 | **m/z** | | 289 | **m/z** | | 881 | **m/z** | | 441 | **m/z** | | 441 | **m/z** | | 577 | **m/z** | | 441 | **m/z** | | 881 |
| PLE-A | SFE-B | 1 | PLE-D | SFE-B | 1 | PLE-G | SFE-B | 1 | PLE-G | SFE-B | 1 | PLE-A | SFE-C | 1 | PLE-H | SFE-C | 1 | PLE-G | SFE-C | 1 | PLE-A | SFE-B | 1 |
|  | SLE-C | 0 |  | SLE-B | 1 |  | SLE-C | 0 |  | SLE-D | 1 |  | SLE-C | 1 |  | SLE-B | 1 |  | SLE-C | 1 |  | SLE-C | 1 |
| SFE-B | PLE-A | 1 | SFE-B | PLE-D | 1 | SFE-B | PLE-G | 1 | SFE-B | PLE-G | 1 | SFE-C | PLE-A | 1 | SFE-C | PLE-H | 1 | SFE-C | PLE-G | 1 | SFE-B | PLE-A | 1 |
|  | SLE-C | 1 |  | SLE-B | 1 |  | SLE-C | 1 |  | SLE-D | 1 |  | SLE-C | 1 |  | SLE-B | 1 |  | SLE-C | 1 |  | SLE-C | 1 |
| SLE-C | PLE-A | 0 | SLE-B | PLE-D | 1 | SLE-C | PLE-G | 0 | SLE-D | PLE-G | 1 | SLE-C | PLE-A | 1 | SLE-B | PLE-H | 1 | SLE-C | PLE-G | 1 | SLE-C | PLE-A | 1 |
|  | SFE-B | 1 |  | SFE-B | 1 |  | SFE-B | 1 |  | SFE-B | 1 |  | SFE-C | 1 |  | SFE-C | 1 |  | SFE-C | 1 |  | SFE-B | 1 |
| **Peak** | | 51 | **Peak** | | 52 | **Peak** | | 53 | **Peak** | | 56 | **Peak** | | 58 | **Peak** | | 63 | **Peak** | | 66 | **Peak** | | 67 |
| **m/z** | | 729 | **m/z** | | 425 | **m/z** | | 479 | **m/z** | | 541 | **m/z** | | 439 | **m/z** | | 329 | **m/z** | | 329 | **m/z** | | 300 |
| PLE-A | SFE-B | 1 | PLE-A | SFE-B | 1 | PLE-B | SFE-B | 0 | PLE-B | SFE-B | 1 | PLE-B | SFE-B | 1 | PLE-B | SFE-B | 1 | PLE-D | SFE-B | 1 | PLE-G | SFE-B | 1 |
|  | SLE-B | 0 |  | SLE-C | 1 |  | SLE-C | 1 |  | SLE-C | 1 |  | SLE-A | 1 |  | SLE-C | 0 |  | SLE-C | 0 |  | SLE-D | 0 |
| SFE-B | PLE-A | 1 | SFE-B | PLE-A | 1 | SFE-B | PLE-B | 0 | SFE-B | PLE-B | 1 | SFE-B | PLE-B | 1 | SFE-B | PLE-B | 1 | SFE-B | PLE-D | 1 | SFE-B | PLE-G | 1 |
|  | SLE-B | 1 |  | SLE-C | 1 |  | SLE-C | 0 |  | SLE-C | 0 |  | SLE-A | 1 |  | SLE-C | 1 |  | SLE-C | 1 |  | SLE-D | 1 |
| SLE-B | PLE-A | 0 | SLE-C | PLE-A | 1 | SLE-C | PLE-B | 1 | SLE-C | PLE-B | 1 | SLE-A | PLE-B | 1 | SLE-C | PLE-B | 0 | SLE-C | PLE-D | 0 | SLE-D | PLE-G | 0 |
|  | SFE-B | 1 |  | SFE-B | 1 |  | SFE-B | 0 |  | SFE-B | 0 |  | SFE-B | 1 |  | SFE-B | 1 |  | SFE-B | 1 |  | SFE-B | 1 |
| 1 - There are significant differences. *p* ≤ 0.05 0 - There are not significant differences | | | | | | | | | | | | | | | | | | | | | | | |

© 2018 by the authors. Submitted for possible open access publication under the terms and conditions of the Creative Commons Attribution (CC BY) license (http://creativecommons.org/licenses/by/4.0/).
